# Response gene to complement 32 expression in macrophages augments paracrine stimulation-mediated colon cancer progression

**DOI:** 10.1038/s41419-019-2006-2

**Published:** 2019-10-10

**Authors:** Peng Zhao, Bing Wang, Zhen Zhang, Wei Zhang, Yan Liu

**Affiliations:** 1 Biotherapy Center, Qingdao Central Hospital, The Second Clinical Hospital of Qingdao University, Qingdao, China; 2 Department of Immunology, Qingdao Universtiy, Qingdao, China; 3 Department of Pathology, The 971 Hospital of People’s Liberation Army Navy, Qingdao, China

**Keywords:** Cancer, Tumour immunology

## Abstract

M2-polarized tumor associated macrophages (TAMs) play an important role in tumor progression. It has been reported that response gene to complement 32 (RGC-32) promotes M2 macrophage polarization. However, whether RGC-32 expression in macrophages could play a potential role in tumor progression remain unclear. Here we identified that increasing RGC-32 expression in colon cancer and tumor associated macrophages was positively correlated with cancer progression. In vitro studies confirmed that colon cancer cells upregulated RGC-32 expression of macrophages via secreting TGF-β1. RGC-32 expression promoted macrophage migration. In addition, stimulation of HCT-116 cells with the condition mediums of RGC-32-silienced or over-expressed macrophages affected tumor cell colony formation and migration via altered COX-2 expression. In an animal model, macrophages with RGC-32 knockdown significantly decreased the expression of COX-2 and Ki67 in the xenografts, and partly inhibited tumor growth. Together, our results provide the evidences for a critical role of TGF-β1/RGC-32 pathway in TAMs and colon cancer cells during tumor progression.

## Introduction

Colorectal cancer (CRC) is the most commonly diagnosed malignancy and the third leading cause of cancer-related death worldwide^1,2^. Despite that CRC screening and therapy have been improved, mortality rates have failed to decrease significantly^3,4^. Tumor associated macrophages (TAMs) are the major inflammatory cellular population in tumor microenvironment^5,6^. High frequencies of TAMs were found in tumor tissues of colon cancer patients^7,8^. Some clinical studies imply an important functional contribution of TAMs to poor prognosis or recurrence of colon cancer^9,10^. It is commonly accepted that TAMs are a distinct M2-polarized population promoting tumor progression, including the promotion of tumor cell proliferation, migration and angiogenesis^11,12^. Thus, TAMs could be potential targets of therapeutic intervention.

Response Gene to Complement (RGC)-32 is a cell cycle regulator involved in cell proliferation, differentiation and immune regulation^13–15^. It is regulated by many growth factors and cytokines, such as VEGF, TGF-β, IL-1β, and TNF-α^16,17^. The over-expression of RGC-32 is found in various types of tumors and regulates tumor progression^18,19^. In addition, the deregulated RGC-32 expression is found in monocytes from patients with Hyper-immunoglobulin E syndrome and multiple sclerosis^20,21^. Importantly, we and Tang, R et al. have demonstrated that RGC-32 is expressed at high levels in M2 macrophages and influences M1/M2 macrophage polarization^22,23^. However, whether altered RGC-32 expression in macrophages contributes to tumor progression in colon cancer microenvironment is unclear.

In this study, we tried to illustrate the regulatory mechanism of RGC-32 in the colon cancer microenvironment and the role of RGC-32 in the progression of colon cancer.

## Results

### RGC-32 expression in human colon cancer tissues

**Fig. 1:**
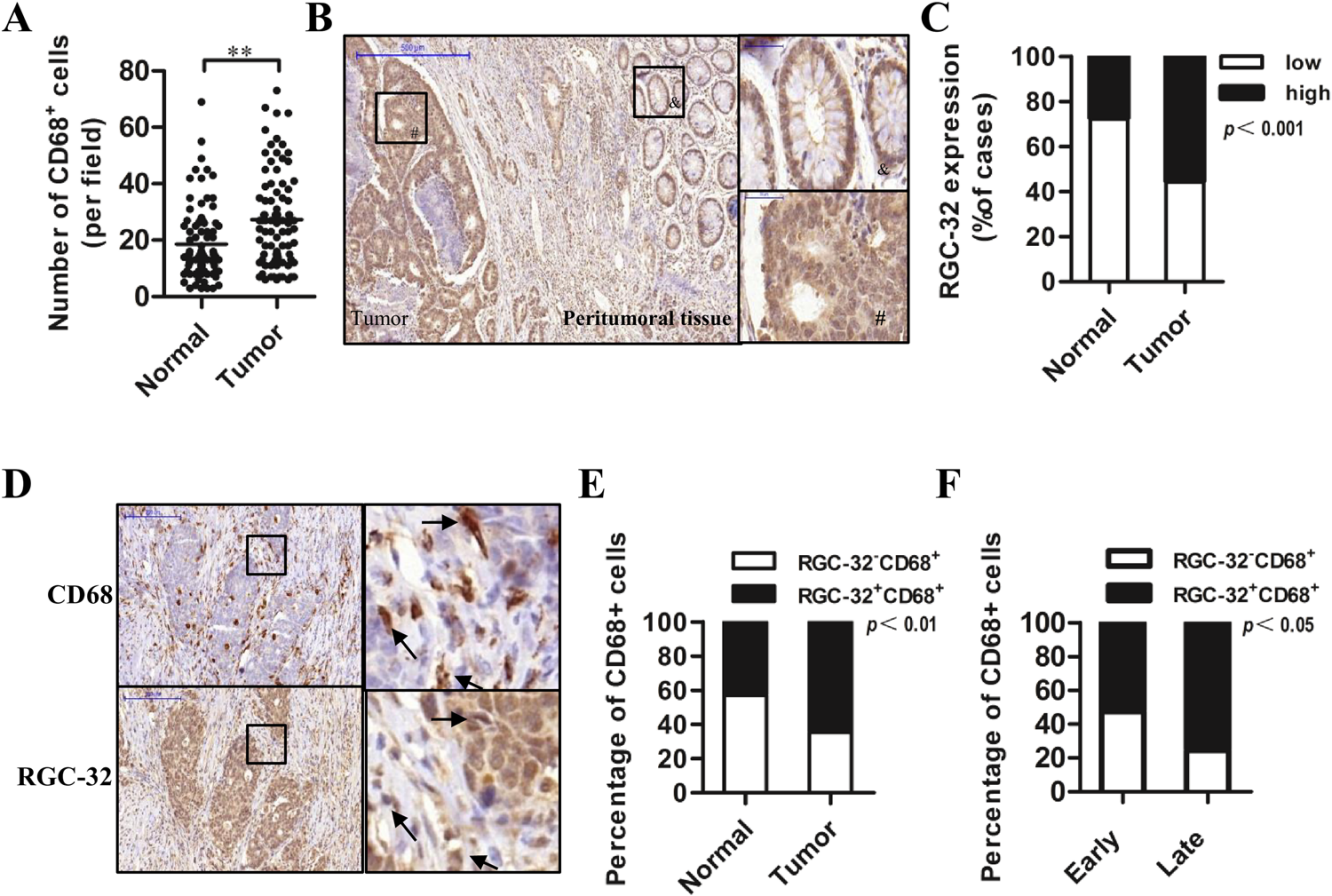
The CD68 and RGC-32 expression in colon cancer tissue. **a** CD68^+^ cell numbers in normal mucosa and tumoral stroma of 79 cases of primary colon cancer. **b** Representative images of RGC-32 expression in peritumoral and tumor tissue in colon cancer (small: 100×; large 400×). **c** The percentage of high-RGC-32 cases in normal and tumor samples. **d** A typical CD68 and RGC-32 expression in two consecutive sections (small: 100×; large 400×). **e** The percentage of samples with RGC-32^+^CD68^+^ macrophages and RGC-32^−^CD68^+^ macrophages in normal and tumor specimens. **f** The percentage of cases with RGC-32^+^CD68^+^ macrophages and RGC-32^−^CD68^+^ macrophages in the early stage and late stage group

To analyze macrophage infiltration into human colon cancer, the immunohistochemistry for macrophage marker CD68 was performed on all samples. Significantly higher numbers of CD68^+^ cells were observed in colon cancer tissue compared with normal colon mucosa (Fig. 1a and Fig. S1a). The number of CD68^+^ macrophages in tumor tissues was positively correlated with TNM stage (Table 1).

**Table 1:** RGC-32 and CD68 expression in relation to clinicopathological parameters of colon cancer patients

	RGC-32 expression			CD68 expression		
Characteristics	Low	High	*P* value	Low	High	*P* value
Age			0.385			0.358
≤60	11	18		11	18	
>60	24	26		24	26	
Gender			0.45			0.848
Male	21	30		23	28	
Female	14	14		12	16	
Differentiation			0.002			0.82
High	16	5		9	12	
Moderate	14	26		19	21	
Low	5	13		7	11	
TNM stage			0.059			0.003
I–II	21	17		24	14	
III–IV	14	27		12	29	
Tumor size			0.705			0.326
≤4 cm	16	22		19	19	
>4 cm	19	22		16	25	
Lymph node			0.034			0.457
0	25	21		22	24	
1	10	23		13	20	
Metastasis			1			0.84
0	34	42		33	43	
1	1	2		2	1	

Next, we explored RGC-32 expression in colon cancer patients. RGC-32 expression was detected as cytoplasmic staining and in some cases RGC-32 was additionally detected as nuclei staining in colon cancer cells. The staining intensity was higher in colon cancer compared with peritumoral (Fig. 1b) or normal tissue (Fig. 1c). The correlation between RGC-32 expression and clinicopathologic parameters was investigated. As shown in Table 1, RGC-32 expression was positively correlated with tumor dedifferentiation and lymph node metastasis. In addition, we found that RGC-32 was expressed in infiltrating macrophages of colon cancer tissues (Fig. 1d). The tissues with RGC-32^+^CD68^+^ macrophages accounted for 64.6% of those with CD68^+^ macrophages in tumor specimens, and only 43% of those in normal tissues (Fig. 1e). As shown in Fig. 1f and Fig. S1b, the percentage of tissues with RGC-32^+^CD68^+^ macrophages in the late stage group (TNM stage III–IV) was notably higher than that in the early stage group (TNM stage I–II). Together, these data suggested that RGC32^+^ macrophages were positively correlated with the progression of colon cancer.

### RGC-32 and CD68 expression in macrophages are associated with poor prognosis of colon cancer patients

**Fig. 2:**
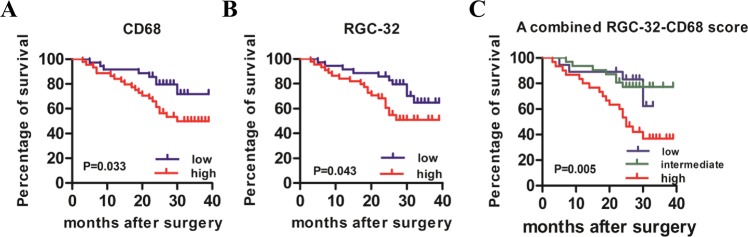
CD68^+^ cells and RGC-32 expression were associated with the survival of colon cancer patients. **a** Kaplan-Meier survival curve revealing the overall survival of patients with low and high CD68. **b** Kaplan-Meier survival curve showed the correlation of overall survival and high or low RGC-32. **c** All patients were divided into three subgroups: RGC-32^−^CD68^low^, intermediate (RGC-32^+^CD68^low^/RGC-32^−^CD68^high^), RGC-32^+^CD68^high^. Overall survival rates of all patients after surgery were analyzed with Kaplan–Meier survival curves

We investigated potential relationships of CD68^+^ macrophages and RGC-32 expression with prognosis. Patients were divided into low/high subgroup for CD68 or RGC-32 as described in the Materials and Methods. Kaplan–Meier survival curve demonstrated that CD68 low group had a significant advantage in survival compared with CD68 high group (Fig. 2a). This survival advantage was also shown in RGC-32 low group (Fig. 2b). Based on RGC-32 and CD68 expression in macrophages, we generated a prognostic score. Colon cancer patients were divided into low (RGC-32^−^CD68^low^), intermediate (RGC-32^−^CD68^high^/RGC-32^+^CD68^low^) and high (RGC-32^+^CD68^high^) groups. As shown in Fig. 2c, patient with high levels of RGC-32 expression and macrophages infiltrating showed statistically significant poor survival. In addition, multivariate Cox regression analyses demonstrated that RGC-32-CD68 prognostic score and tumor differentiation were independent prognostic predictors for overall survival (Table 2).

**Table 2:** Cox proportional hazard model in 79 colon cancer cases

Variable	HR	95% CI	*P* value
Differentiation	1.857	1.071–3.22	0.028
TNM stage	1.648	0.508–5.344	0.405
Tumor size	1.871	0.806–4.34	0.145
Lymph node	0.909	0.322–2.563	0.857
RGC-32-CD68 prognostic score	1.833	1.038–3.239	0.037

### RGC-32 expression in TAMs was induced by TGF-β1 from colon cancer cells

**Fig. 3:**
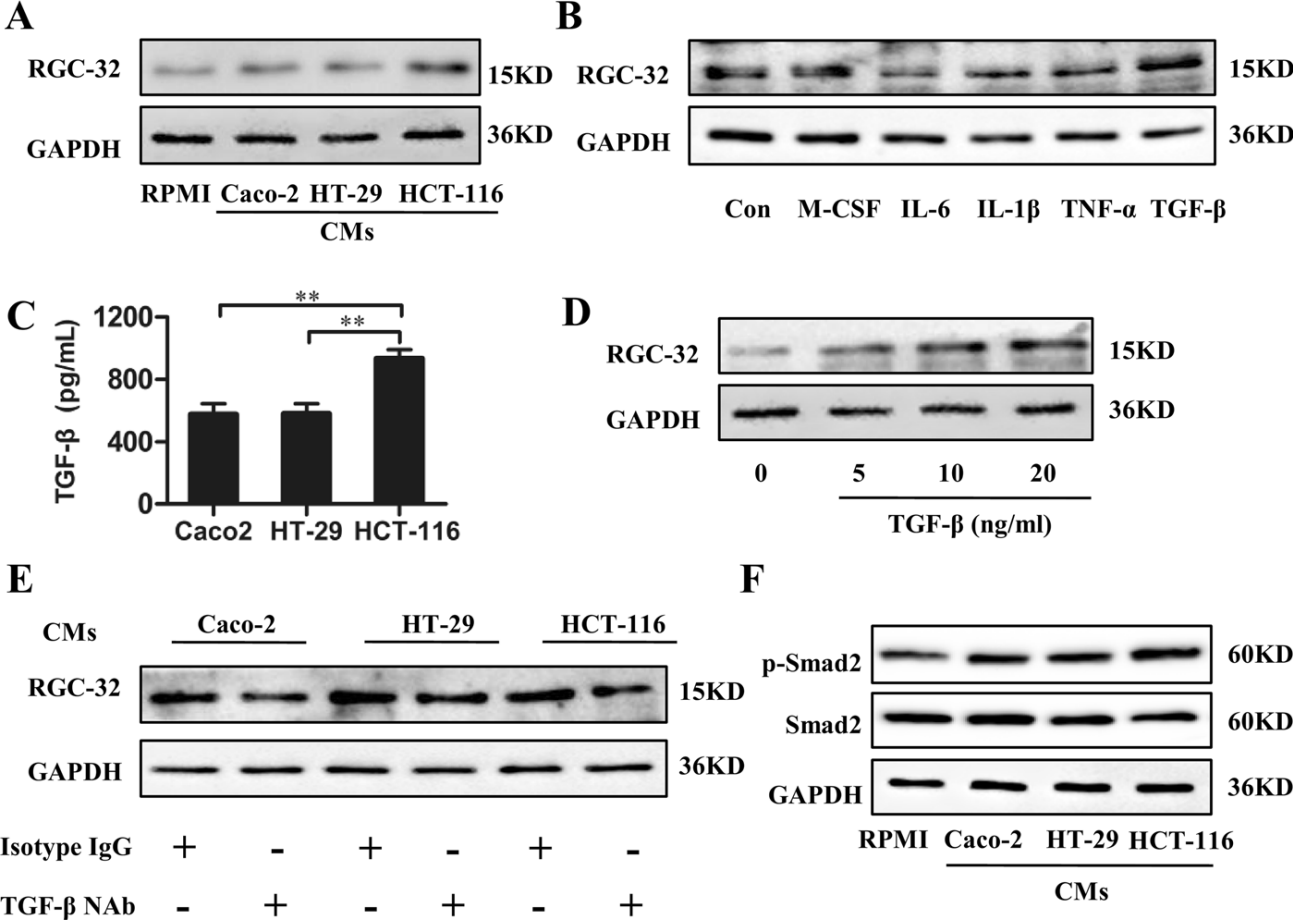
TGF-β1 from colon cancer cell lines increased RGC-32 expression in THP-1 macrophages. **a** Western-blot analysis of RGC-32 protein in macrophages stimulated by PMA and the CMs of HT-29, Caco-2 and HCT-116 cells. **b** RGC-32 protein in macrophages stimulated by PMA plus M-CSF (20 ng/ml), IL-6 (10 ng/ml), IL-1β (10 ng/ml), TNF-α (10 ng/ml), and TGF-β (10 ng/ml) was determined, respectively. **c** HT-29, Caco-2, and HCT-116 cells were cultured for 96 h, and then the culture medium were harvested and measured by ELISA. **d** THP-1 cells were stimulated with PMA and graded dose of TGF-β1. RGC-32 expression in THP-1 macrophages was determined by western blotting. **e** THP-1 cells were induced by PMA and tumor cell-CMs pretreated with/without TGF-β1 NAb for 48 h. RGC-32 expression in THP-1 macrophages was determined by western blotting. **f** The phosphorylation of Smad2 in THP-1 cells induced by PMA and the CMs of colon cancer cell lines was determined through western blot analysis

In the above study, we found that the percentage of tissues with RGC-32^+^CD68^+^ macrophages was significantly increased in tumor tissue. To determine whether RGC-32 expression was induced by the tumor microenvironment, additional treatment with the Condition mediums (CMs) of Caco2, HT-29, and HCT-116 cells in the presence of Phorbol 12-myristate 13-acetate (PMA) induced the differentiation of monocyte cell line THP-1 cells into macrophages. Our results showed that RGC-32 protein level in THP-1 macrophages was upregulated in response to stimulation with the CMs of three colon cancer cell lines, with the highest levels induced by HCT-116 cells (Fig. 3a).

A panel of cytokines, M-CSF, IL-6, IL-1β, TNF-α, and TGF-β, were incubated with THP-1 cells in the presence of PMA for 48 h, and RGC-32 level was determined. As shown in Fig. 3b, TGF-β1 but not M-CSF, significantly induced RGC-32 expression, whereas IL-6, IL-1β, and TNF-α decreased RGC-32 expression. TGF-β1 was produced by three colon cancer cells, with the highest amounts being produced by HCT-116 cells (Fig. 3c). TGF-β1-induced RGC-32 expression in THP-1 macrophages showed the dose dependence (Fig. 3d), indicating that TGF-β1 might be play an important role in RGC-32 expression induced by the CMs of colon cancer cell lines. To test the hypothesis, the CMs of colon cancer cells were pretreated with TGF-β1 NAb, and then induced the differentiation of THP-1 cell into macrophages. RGC-32 expression of THP-1 macrophages was significantly blocked by TGF-β1 NAb (Fig. 3e). We then investigated the changes in downstream molecules of TGF-β signaling pathway. The results showed that the treatment with the CMs of colon cancer cells significantly increased the phosphorylation of smad2 in THP-1 macrophages (Fig. 3f). Thus, TGF-β1 secreted by colon cancer cells is involved in the regulation of RGC-32 expression in THP-1 macrophages.

### RGC-32 promoted macrophage migration

**Fig. 4:**
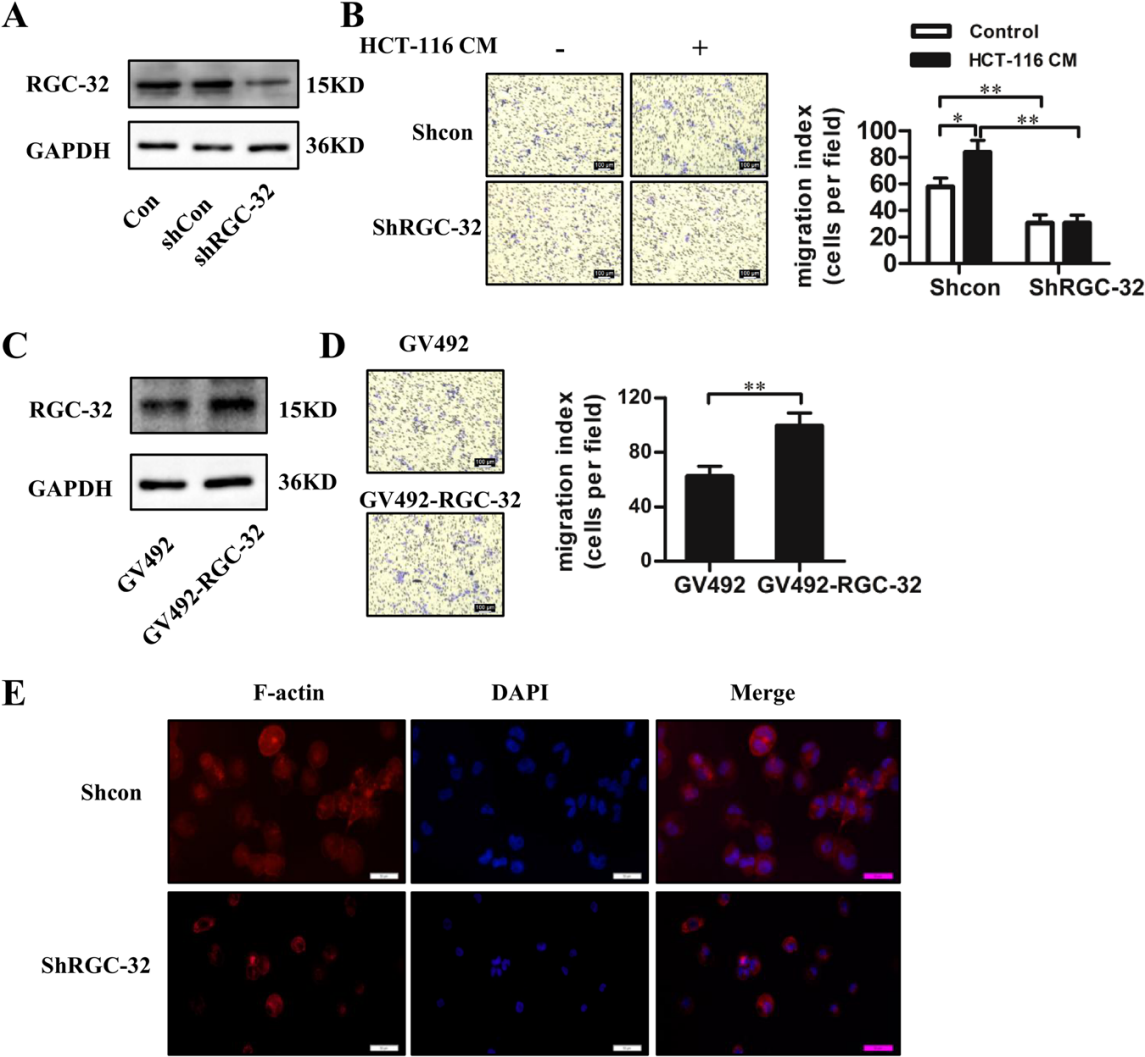
The effect of RGC-32 expression on the migration of THP-1 macrophages. THP-1 cells were transfected with RGC-32 shRNA and shRNA controls or GV492 and GV492-RGC-32. The transfected cells were differentiated into THP-1 macrophages. **a**, **c** RGC-32 expression in THP-1 macrophages was detected by western blotting. **b** Stimulation of HCT-116 CM significantly increased the migration of THP-1 macrophages. Knockdown of RGC-32 in THP-1 macrophages significantly decreased cell migration compared to shRNA control. **d** Transwell assay showed that the migration was accelerated in RGC-32 over-expressed macrophages compared to control cells. **e** Fluorescent staining for F-actin (red) with Phalloidin-TRITC in shRGC-32 and shcon macrophages. Data are presented as the mean ± SD (*p < 0.05, **p < 0.01)

The function of elevated RGC-32 expression in TAMs was further investigated. As RGC-32 was reported to be involved in cell migration^24^, we explored the effect of RGC-32 on macrophage migration. RGC-32 shRNA was transfected into THP-1 cells. These modified THP-1 cells were differentiated into macrophage-like cells by stimulation with PMA or plus HCT-116-CM for 48 h (Fig. 4a). Our results showed that RGC-32 knockdown resulted in significant inhibition of macrophage migration compared with control cells when stimulated with or without HCT-116-CM (Fig. 4b). Complementary experiments in which RGC-32 was over-expressed in THP-1 macrophages (Fig. 4c) significantly enhanced cell migration as compared with those infected by lentivirus vector only (Fig. 4d).

Cell migration is usually associated with the changes in the actin cytoskeleton organization^25,26^. Phalloidin-TRITC staining of THP-1 macrophages indicated a disordered structure of cytoskeleton in RGC-32-silenced macrophages (Fig. 4e). In addition, we found that altered RGC-32 expression did not affect the expression of M1/M2 polarization markers CD68, CD163, and CD206 (data not shown). These data suggest that RGC-32 facilitates the migration of macrophages, which might promote macrophage infiltration into tumor tissue.

### RGC-32 expression in colon cancer cells promotes tumor cell proliferation and migration

We found that RGC-32 was over-expressed in colon cancer tissues and correlated with tumor progression. To assess the direct effect of RGC-32 on cancer cells, lentiviral vectors targeting or over-expressing RGC-32 were transfected into HCT-116 cells. Then we performed CCK8 and transwell assay to evaluate the effect of RGC-32 on cell proliferation and migration. RNA interference suppression of RGC-32 in HCT-116 cells reduced cell proliferation and migration of HCT-116 cells compared with the shRNA controls (Fig. S2). Complementary experiments in which RGC-32 was over-expressed in HCT-116 cells resulted in significant elevation of cell proliferation and migration (Fig. S2).

### RGC-32 expression in macrophages promotes the colony formation and migration of colon cancer cells

**Fig. 5:**
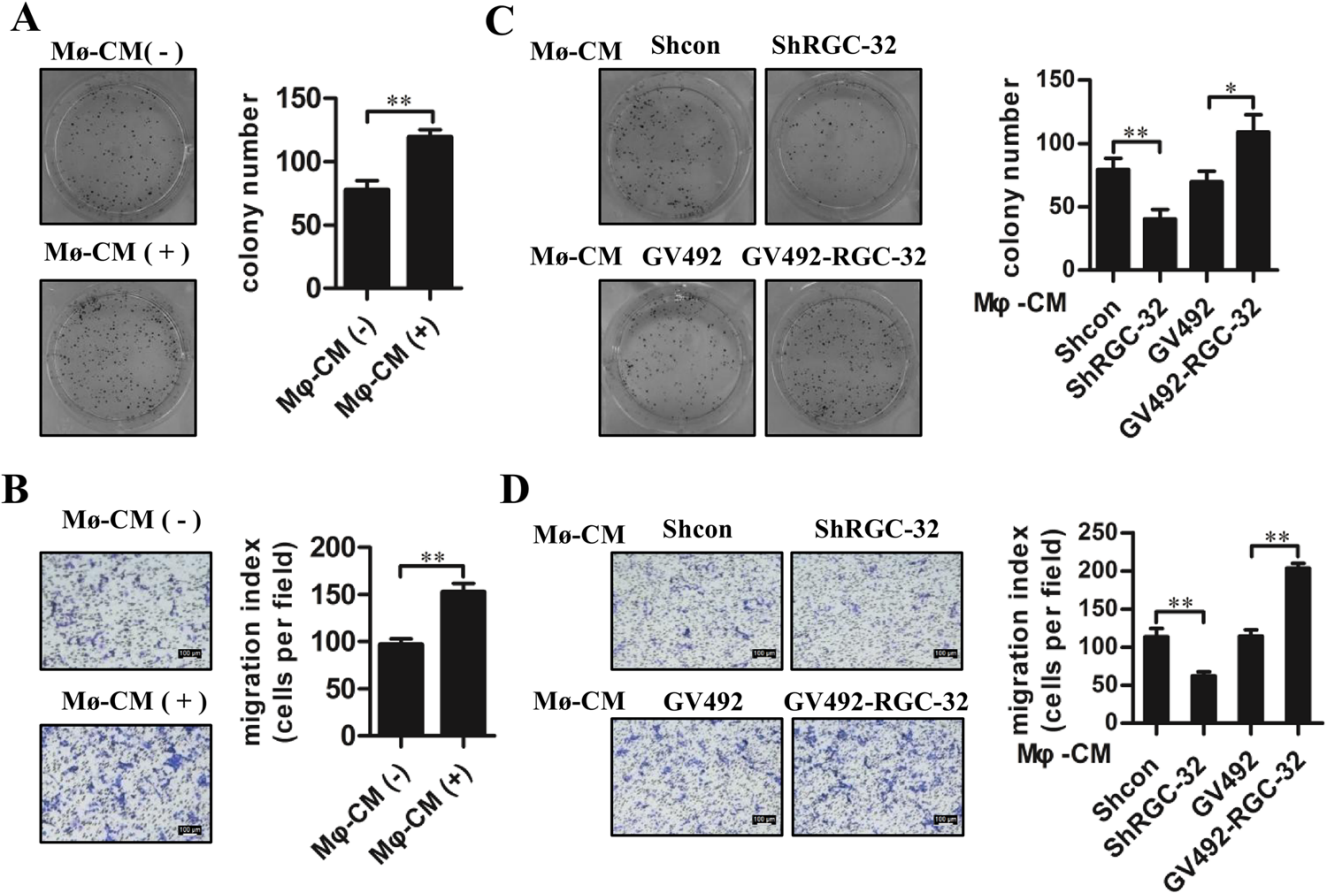
RGC-32 expression in macrophages regulated the colony formation and migration of HCT-116 cells. **a**, **b** HCT-116 cells were stimulated with the CMs of THP-1 macrophages for 48 h. Tumor cell colony formation and migration were determined. **c**, **d** HCT-116 cells were stimulated with the CMs of shRGC-32 and shcon-transfected THP-1 macrophages or GV492-RGC-32 and control vector-transfected THP-1 macrophages for 48 h. Tumor cell colony formation and migration were determined. Each bar represents the mean ± SD (*p < 0.05, **p < 0.01)

To study the effects of macrophages on tumor progression, we measured the effects of THP-1 macrophage-CMs on tumor cell colony formation and migration. As shown in Fig. 5a, b, macrophages-CMs significantly enhanced colony-forming ability and migration of HCT-116 cells. HCT-116 cells that received the CMs derived from THP-1 macrophages with RGC-32 silencing showed diminished clonogenicity and migration than these cells stimulated with the CMs from THP-1 macrophages with shcon treatment (Fig. 5c, d). However, tumor cell colony formation and migration were increased when HCT-116 cells were exposed to CMs from THP-1 macrophages with RGC-32 over-expression (Fig. 5c, d).

### RGC-32 expression in macrophages mediated induction of COX-2 in colon cancer cells

**Fig. 6:**
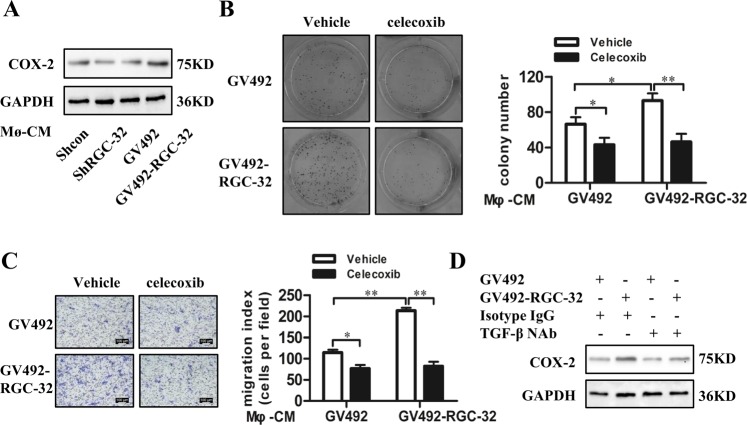
RGC-32 expression in macrophages increased COX-2 expression in colon cancer cells. **a** COX-2 expression in HCT-116 cells was affected by the CMs of shRGC-32 and shRNA-transfected THP-1 macrophages or GV492-RGC-32 and GV492-transfected THP-1 macrophages. **b**, **c** HCT-116 cells were pretreated with celecoxib (10 μM), and then were stimulated by CMs from GV492-RGC-32 and GV492-transfected macrophages, respectively. The cell colony formation and migration of HCT-116 cells were determined. **d** TGF-β1 NAb inhibited COX-2 expression of HCT-116 cells stimulated by the CMs of THP-1 macrophage. All data are shown as the mean ± SD. *p < 0.05, **p < 0.01

Increased expression of cyclooxygenase-2 (COX-2) is a crucial factor in the tumorigenic process^27,28^. Our results showed that COX-2 expression was increased in HCT-116 cells exposed to the CM of macrophages with RGC-32 over-expression and reduced in HCT-116 cells stimulated by the CM of RGC-32-silenced macrophage (Fig. 6a). Next, HCT-116 cells pretreated with a selective COX-2 inhibitor, celecoxib, were exposed to the CMs from THP-1 macrophages to assess the effect on the ability of colony formation and migration. The positive effect of the CMs from RGC-32 over-expressed macrophages on tumor cell colony formation and migration were blocked by celecoxib (Fig. 6b, c).

TGF-β1 is responsible for the induction of COX-2 expression^29,30^. Previous studies have demonstrated that RGC-32 over-expression in macrophages produces more TGF-β^23^. To verify whether the CMs of RGC-32 over-expressed macrophages induced higher level of COX-2 expression in HCT-116 cells through TGF-β1, the CMs of THP-1 macrophages were pretreated with TGF-β1 NAb, and then were incubated with HCT-116 cells. As shown in Fig. 5d, TGF-β1 NAb significantly reduced COX-2 expression in HCT-116 cells stimulated by the CMs of RGC-32 over-expressed macrophages and control cells, whereas it did not completely block the positive effect of the CMs of RGC-32 over-expressed macrophages on COX-2 expression in HCT-116 cells.

### RGC-32 knockdown in macrophages decreases COX-2 and Ki67 expression in the xenograft and partially suppresses tumor growth

**Fig. 7:**
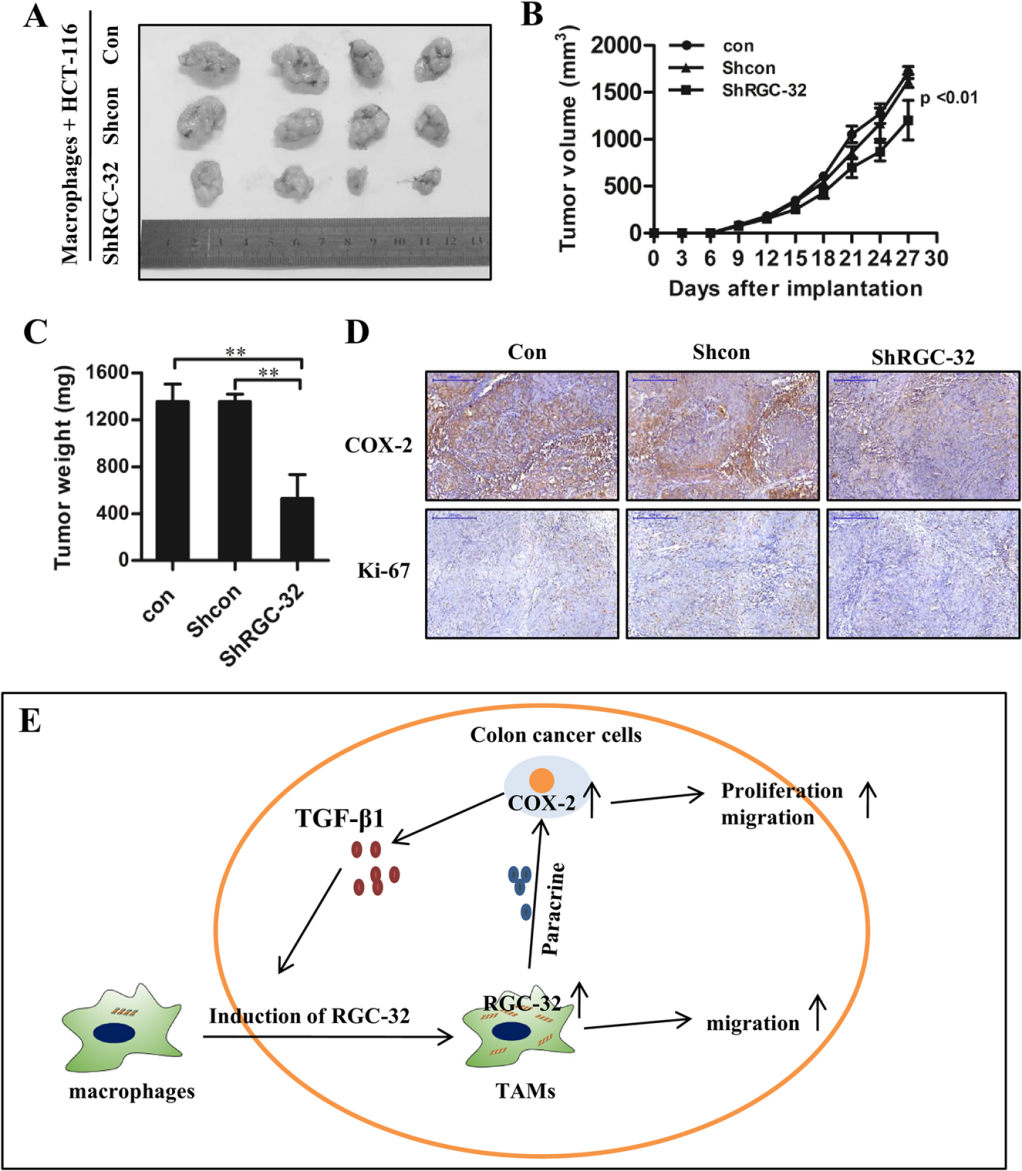
RGC-32 silencing in macrophages inhibited tumor growth, decreased COX-2 and Ki67 expression of xenografts. HCT-116 cells were co-injected with RGC-32-silenced macrophages or control macrophages to establish a subcutaneous tumor model. **a**–**c** Tumor sizes and weights were smaller after co-injection of RGC-32-silenced macrophages and HCT-116 cells as compared with that after co-injection of control macrophages and HCT-116 cells. **d** A lower expression of COX-2 and Ki67 was detected in tumor from co-injection of RGC-32-silenced macrophages and HCT-116 cells (100×). **e** Schematic representation of interactions between colon cancer cells and macrophages through RGC-32. Each bar represents the mean ± SD (*p < 0.05, **p < 0.01)

The effect of RGC-32 expression in macrophages on tumor growth was validated in vivo. HCT-116 cells were co-injected with RGC-32-silenced macrophages (shRGC-32) or control macrophages (shcon or con) into nude mice subcutaneously, respectively. RGC-32-silenced macrophages significantly inhibited tumor volume of xenografts compared with shcon and con groups (Fig. 7a, b). The tumor weight analysis was consistent with tumor volume (Fig. 7c). Immunostaining of HCT-116 cells in the xenografts revealed that the expression of COX-2 and the proliferating antigen Ki67 was also markedly reduced in shRGC-32 group (Fig. 7d).

## Discussion

Macrophages are recruited into the tumor microenvironment and interact with colon cancer cells to facilitate tumor growth and metastasis. However, the mechanism by which tumor-infiltrating macrophages favor tumor development remains unclear. In this study, we investigated the expression and regulation of RGC-32 in the tumor microenvironment and demonstrated a RGC-32-mediated crosstalk mechanism between macrophages and colon cancer cells (Fig. 7e).

RGC-32, a cell cycle regulator, is highly expressed in many tumors and promotes tumor cell proliferation^31–33^. To the contrary, some study has reported that RGC-32 protein is directly induced by P53 and suppresses tumor cell proliferation^34^. In the study, we found RGC-32 over-expression in colon cancer tissues, which was associated with tumor progression and predicted poor prognosis. In vitro studies showed that RGC-32 expression promoted the proliferation and migration of colon cancer cells, indicating that RGC-32 is a tumor-promoting gene in colon cancer cells.

RGC-32 is also reported to express abnormally in immune cells, which is involved in immune disorder^35,36^. Our data showed that the percentage of tissues with RGC-32^+^CD68^+^ cells was higher than those in normal tissue, reflecting that RGC-32 expression might be regulated by tumor microenvironment. TGF-β1 as a multifunctional cytokine is involved in pathological processes, such as inflammatory response and tumorigenesis^37–39^. RGC-32 is a downstream target of TGF-β that mediates epithelial-mesenchymal transition and Th17 cell differentiation^40,41^. In this study, our data indicated that RGC-32 expression in macrophages was significantly increased by colon cancer cell-derived TGF-β1. In addition, we confirmed that RGC-32 expression promoted macrophage cell migration, which might be involved in high macrophage infiltrating into the tumor tissue.

Increasing evidences show that macrophages are recruited into tumor microenvironment and polarized to M2 type to promote tumor progression^42–44^. Previous studies suggest that RGC-32 inhibits M1 phenotype and promotes M2 polarization. In this study, RGC-32 expression in TAMs was significantly correlated with the progression of colon cancer. In vitro studies showed that stimulation of CMs from THP-1 macrophages with RGC-32 over-expression increased the clonogenic formation and migration of HCT-116 cells, and fostered COX-2 expression. Increased expression of COX-2 is a crucial factor in the tumorigenic process. COX-2 and its enzymatic product prostaglandin E_2_ stimulates cancer cell proliferation, inhibits apoptosis and increases metastatic potential^28,45^. We found that the positive effect of the CMs of macrophages with RGC-32 over-expression on the clonogenic formation and migration of HCT-116 cells was inhibited by celecoxib, indicating that RGC-32 expression in TAMs leads to tumor progression via upregulating COX-2 expression. In an animal model, we further demonstrated that RGC-32-silenced macrophages significantly reduced the expression of COX-2 and Ki67 in the xenografts, and partly inhibited tumor growth.

The expression of COX-2 is induced at the site of inflammation in response to inflammatory stimuli, such as IL-1β, TNF-α, TGF-β^46,47^. Our results indicated that TGF-β1 NAb signficantly reduced COX-2 expression in HCT-116 cells exposed to the CMs of THP-1 macrophages. However, the positive effect of the CM of RGC-32 over-expressed macrophages on COX-2 expression was not completely blocked by TGF-β1 NAb, indicating that RGC-32 might influence COX-2 expression in colon cancer through regulating multiple paracrine factors of macrophages.

In conclusion, our results showed that colon cancer cells promoted RGC-32 expression in macrophages, which subsequently enhanced macrophage migration and promoted tumor progression through paracrine mechanisms. Therefore, targeting TGF-β/RGC-32 pathway may exert some antitumor effect.

## Materials and methods

### Antibodies and reagents

PMA was obtained from Sigma (P1585). Anti-COX-2 and anti-CD68 antibodies were purchased from R&D Systems (AF4198 and MAB20401). Rabbit polyclonal anti-RGC-32 antibody was purchased from Biorbyt (orb2372). Anti-smad2 and anti-p-smad2 were purchased from Cell Signaling Technology (5339 and 18338). TGF-β1 neutralizing antibody (NAb) was obtained from R&D Systems (MAB240) and used at 2.5 μg/ml.

### Patients and clinical samples

Tumor specimens of 79 patients diagnosed with primary colon cancer were collected in 971 Hospital of People’s Liberation Army from June 2015 to November 2016. Informed consent for this study was obtained from each patients and the protocol was approved by the Ethics Committee of 971 Hospital of People’s Liberation Army.

### Immunohistochemistry

All tissues were fixed with 4% neutral formaldehyde, and embedded in paraffin for immunohistochemical staining. Specimens were incubated with rabbit polyclonal anti-RGC-32 antibody (1:100) and anti-CD68 antibodies (1:200) at 4 °C overnight and then incubated with second antibodies at 37 °C for 1 h. The sections were stained with 3, 3′-diaminobenzidine and then counterstained with hematoxylin. The samples were visualized by bright field microscopy. Positive and negative controls were used in this study.

The staining intensity of colon cancer cells was scored as negative (0), weak (1), moderate (2), and strong (3). The percentages of positively stained cells were graded as follows: <5% (0), 5–25% (1), 25–50% (2), 50–75% (3), ≥75% (4). The stained intensity and percentage of positive cells were multiplied to generate a final score for each specimen. Tumor specimens were divided into low (0–4) and high (6–12) group according to the intensity scores.

For CD68 evaluation in primary tumor samples, 5 representative areas with high densities (>5 cells/field) of CD68 marker accumulation were selected for each slide, and the numbers of CD68^+^ cells in each tissue core were directly counted. All patients were divided into 2 group based on the median number of CD68^+^ cells infiltrated in primary samples. All cases with the number ≤26 per field were considered low, the number >26 considered high.

### Cell cultures

Colon cancer cell line HCT-116, HT-29, Caco-2, and Human monocytic leukemia cell line THP-1 (purchased from Shanghai Cell Bank, Chinese Academy of Sciences) were cultured in RPMI-1640 medium containing 10% fetal bovine serum (FBS) in an atmosphere containing 5% CO2 at 37 °C.

To generate THP-1 macrophages, THP-1 cells were treated with 100 ng/ml PMA for 48 h. Condition mediums (CMs) were collected from colon cancer cells and THP-1 macrophages after 96 h. THP-1 cells were treated with the CMs of colon cancer cells (30%) supplemented with PMA for 48 h. Macrophages-derived CM was used to stimulate HCT-116 cells for 48 h.

### Lentivirus infections

THP-1 cells were transfected with two short hairpin RNAs (shRNA) targeting RGC-32 using lentivirus vector GV248 (Genechem, Shanghai, China). The shRNA sequences were as follows: CACTCCTCAGAAAGCTAAA and ACAGACGATCCATGCTAAT. The RGC-32 over-expressing cell line were generated by lentivirus vector GV492 containing the full length sequence of RGC-32 (Genechem, Shanghai, China). The efficiency of RGC-32 knockdown and over-expression was validated by western blotting.

### Phalloidin-TRITC staining

ShRGC-32 or shcon THP-1 macrophages were seeded on glass coverslips in 24-well plates and cultured for 24 h. The cells were then fixed with 4% formaldehyde, permeabilized using 0.1% Triton X-100, and F-actin was fluorescently labeled using 0.1 μg/ml Phalloidin-TRITC (Sigma-Aldrich). Nuclei were stained using DAPI. Images were captured using an inverted fluorescence microscope and adjusted using Adobe Photoshop software.

### Western-blot

THP-1 macrophages or colon cancer cell lines were lysed in RIPA buffer containing a protease inhibitor cocktail. Cell lysates were subjected to electrophoresis on 10% SDS-polyacrylamide gels and transferred onto a nitrocellulose membrane. After blocking at room temperature for 1 h with TBST (Tris-buffered saline, 0.1% Tween-20) containing 5% non-fat dried milk, the membranes were incubated with the indicated antibodies, including anti-RGC-32, anti-smad2, anti-p-smad2, anti-COX-2, and anti-GAPDH overnight at 4 °C. The next day, membranes were washed with TBST and incubated with HRP-conjugated anti-mouse or anti-rabbit IgG for 1 h. The protein bands were detected with enhanced chemiluminescence.

### Transwell assay

1 × 10^5^ cells/well THP-1 macrophages or HCT-116 cells were added into the upper chamber of the inserts in 200 μl of serum-free medium. Six hundred microliter RPMI-1640 medium containing 20% FBS was added into the lower chamber. Cells were incubated at 37 °C in 5% CO_2_ for 24 h. The migrated cells on the lower side of the filter were fixed by methanol and stained with Giemsa. Five random fields of each well were photographed and cell numbers were counted.

### Colony formation assay

Colon cancer cells were transfected with lentivirus vector, or treated with the CMs from THP-1 macrophages. Cells were trypsinized, counted and replated at a density of 500 cells/well into a 6-well plate. Ten days later, colonies resulting from the surviving cells were fixed with methanol, stained with Giemsa and counted. Colonies containing at least 50 cells were scored. Each assay was performed in triplicates.

### Cytokine measurement

The medium of colon cancer cell line HT-29, Caco-2, and HCT-116 were collected. TGF-β1 was determined by ELISA kits (R&D Systems, DB100B) according to the manufacturer’s instruction.

### Animal experiments

All animal experiments were approved by the Institutional Animal Care and Use Committee at Qingdao Central Hospital. RGC-32-silenced (shRGC-32)/control (shcon or con) macrophages and HCT-116 cells were mixed at a ratio of 1:4 and a total of 5 × 10^6^ cells were inoculated subcutaneously into the right leg of five-week-old male BALB/c nude mice. Tumor growth was evaluated by monitoring tumor volume every 3 days for 4 weeks. The animals were sacrificed and tumors were isolated and weighted. Immunohistochemistry was performed to detect the expression of COX-2 and Ki67.

### Statistic analysis

Data are presented as mean ± SD based on triplet experiments. Statistical comparisons among groups were made by Student’s *t*-test and ANOVA. Spearman’s test was used for the correlation analysis. Kaplan–Meier analysis was used and *P*-values were determined by the log–rank test. Factors associated with overall survival with a *p* value lower than 0.05 were further tested in the multivariate analysis by the Cox model. Statistical significance was considered *p* < 0.05.

## Supplementary information

Supplementary figure legends

Figure S1 Representative images of colon cancer samples immunostained for CD68 and RGC-32

Figure S2 RGC-32 expression in HCT-116 cells promotes tumor cell proliferation and migration
