# Supplementary material for: Response gene to complement 32 expression in macrophages augments paracrine stimulation-mediated colon cancer progression
Source: Cell Death Dis. 2019 Oct 10;10(10):776. doi: 10.1038/s41419-019-2006-2 (PMC6786990; doi:10.1038/s41419-019-2006-2)
Supplement: Supplementary file 1 — Supplementary figure legends [file 41419_2019_2006_MOESM1_ESM.doc]

**Figure S1** Representative images of colon cancer samples immunostained for CD68 and RGC-32. (**A**) Representative immunohistochemical staining of CD68 in normal and tumor tissues (100×). (**B**) Immunohistochemical double staining of CD68/RGC-32 for the early and late specimens. CD68 was stained with red and RGC-32 was stained with yellow. RGC-32^-^CD68^+^ macrophages were shown by black arrows and RGC-32^+^CD68^+^ macrophages were shown by green arrows (small: 100×; large 200×).

**Figure S2** RGC-32 expression in HCT-116 cells promotes tumor cell proliferation and migration. Colon cancer cells were transfected with shRGC-32 and Shcon or GV492-RGC-32 and control vector. (**A**) Cell viability was evaluated using the CCK8 assay. (**B**) Cell migration was evaluated by a Transwell assay. Each bar represents the mean ± SD (*p < 0.05, **p < 0.01).
