# Supplementary figures and images for: Response gene to complement 32 expression in macrophages augments paracrine stimulation-mediated colon cancer progression
Source: Cell Death Dis. 2019 Oct 10;10(10):776. doi: 10.1038/s41419-019-2006-2 (PMC6786990; doi:10.1038/s41419-019-2006-2)

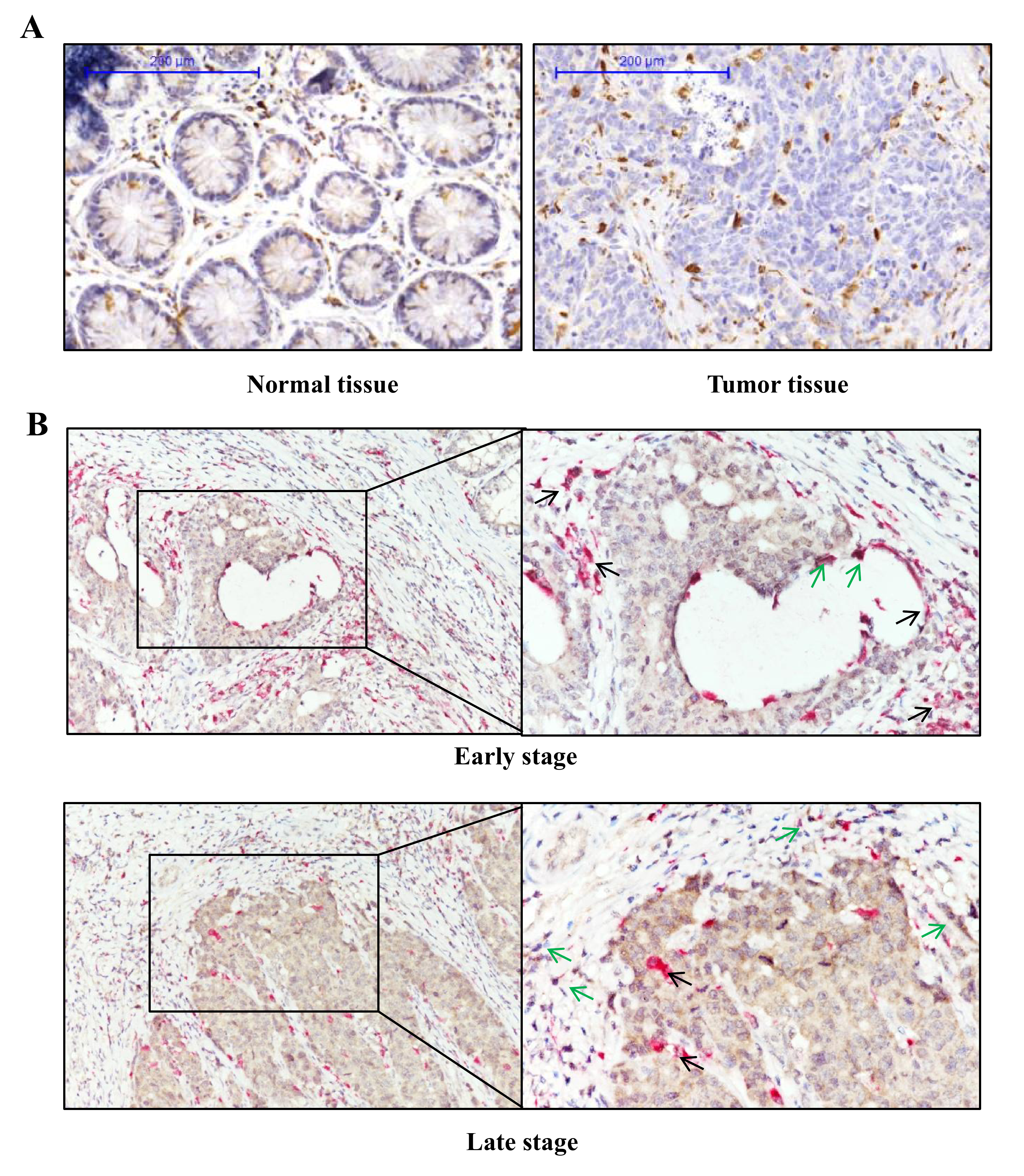

Supplement: Supplementary file 2 — Figure S1 Representative images of colon cancer samples immunostained for CD68 and RGC-32 [file 41419_2019_2006_MOESM2_ESM.tif]

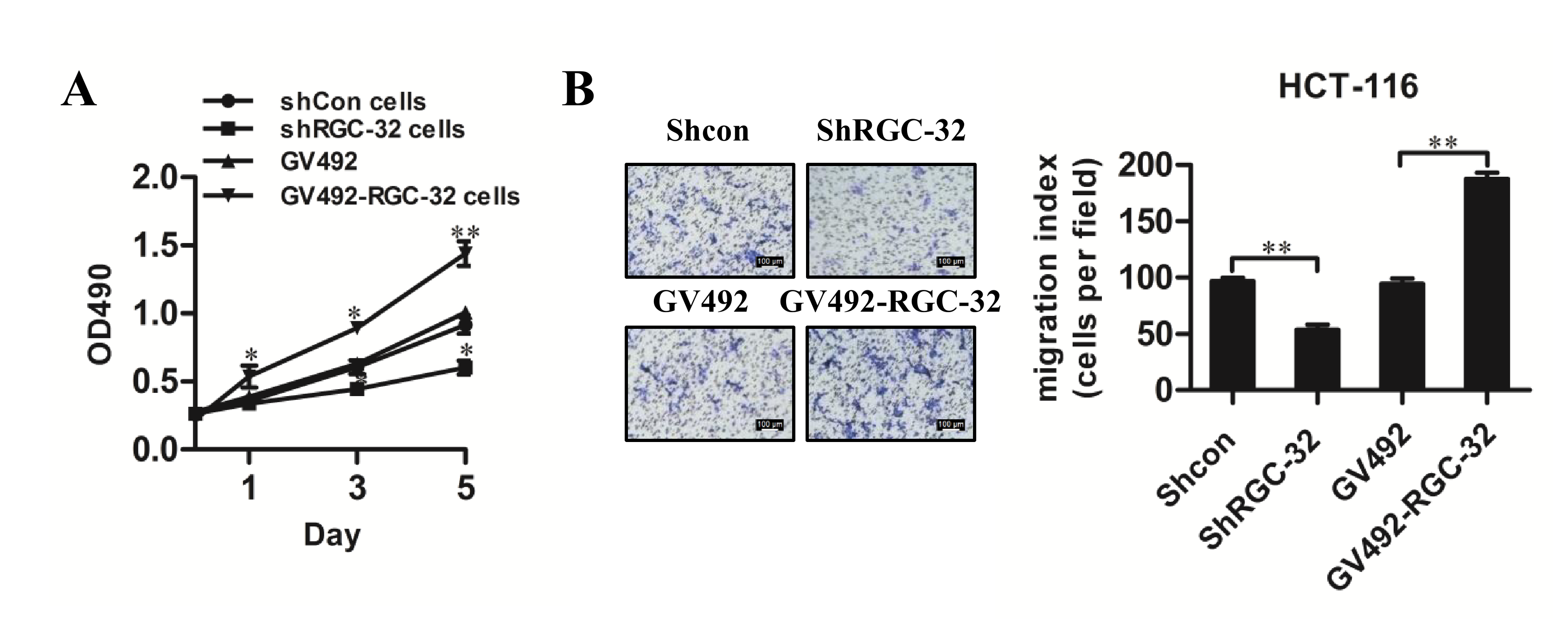

Supplement: Supplementary file 3 — Figure S2 RGC-32 expression in HCT-116 cells promotes tumor cell proliferation and migration [file 41419_2019_2006_MOESM3_ESM.tif]
